# Supplementary material for: Transcriptomics-Based Drug Repurposing Approach Identifies Novel Drugs against Sorafenib-Resistant Hepatocellular Carcinoma
Source: Cancers (Basel). 2020 Sep 23;12(10):2730. doi: 10.3390/cancers12102730 (PMC7598246; doi:10.3390/cancers12102730)
Supplement: Supplementary file 1 [file cancers-12-02730-s001.zip › cancers-887161 Supplementary Figure S1-S6-proofread.pdf]

Supplementary Materials

Transcriptomics-Based Drug Repurposing Approach Identifies Novel Drugs against Sorafenib-Resistant Hepatocellular Carcinoma

Kelly Regan-Fendt, Ding Li, Ryan Reyes, Lianbo Yu, Nissar A. Wani, Peng Hu, Samson T. Jacob , Kalpana Ghoshal, Philip R. O. Payne and Tasneem Motiwala

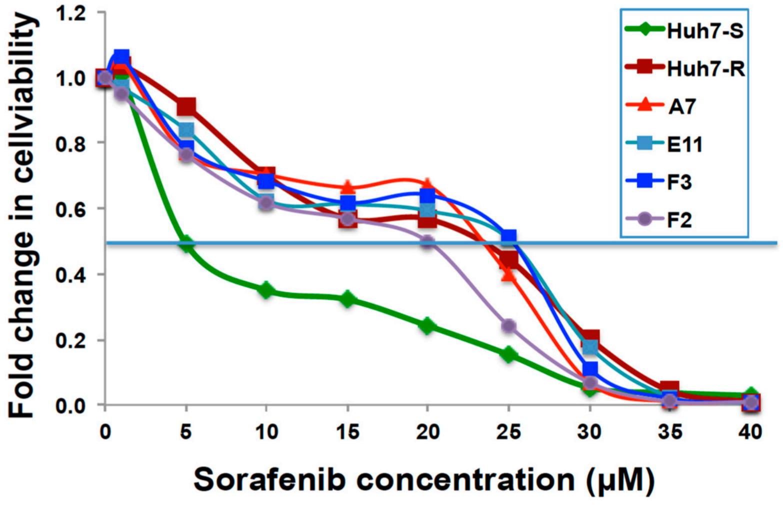

Figure S1. Sorafenib dose response curve for sorafenib sensitive and resistant Huh7 cells. Fold-change in cell viability of Huh7 cells treated with increasing concentrations of sorafenib, as measured 48 hours post-treatment using an ATP assay (CellTiter-Glo). The blue horizontal line marks the IC_{50} values. Huh7-S: parental Huh7 cells; Huh7-R: pool of sorafenib-resistant cells; A7, E11, F3, F2: individual sorafenib-resistant clones. Increased IC_{50} values are demonstrated for the sorafenib-resistant pool and individual clones relative to parental cells.

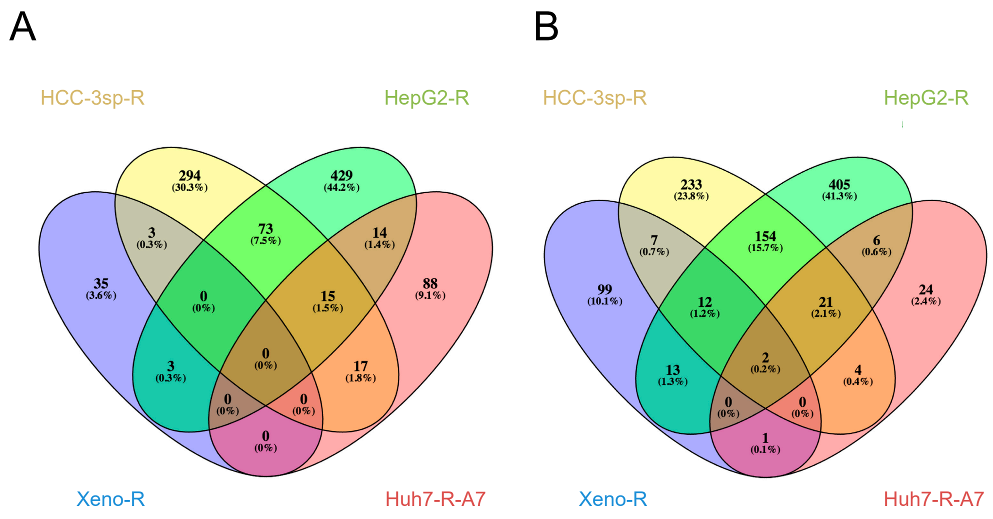

Figure S2. Comparison of HCC sorafenib resistance gene signatures. Overlap of up-regulated (**A**) and down-regulated (**B**) genes among the four HCC sorafenib resistance gene signatures.

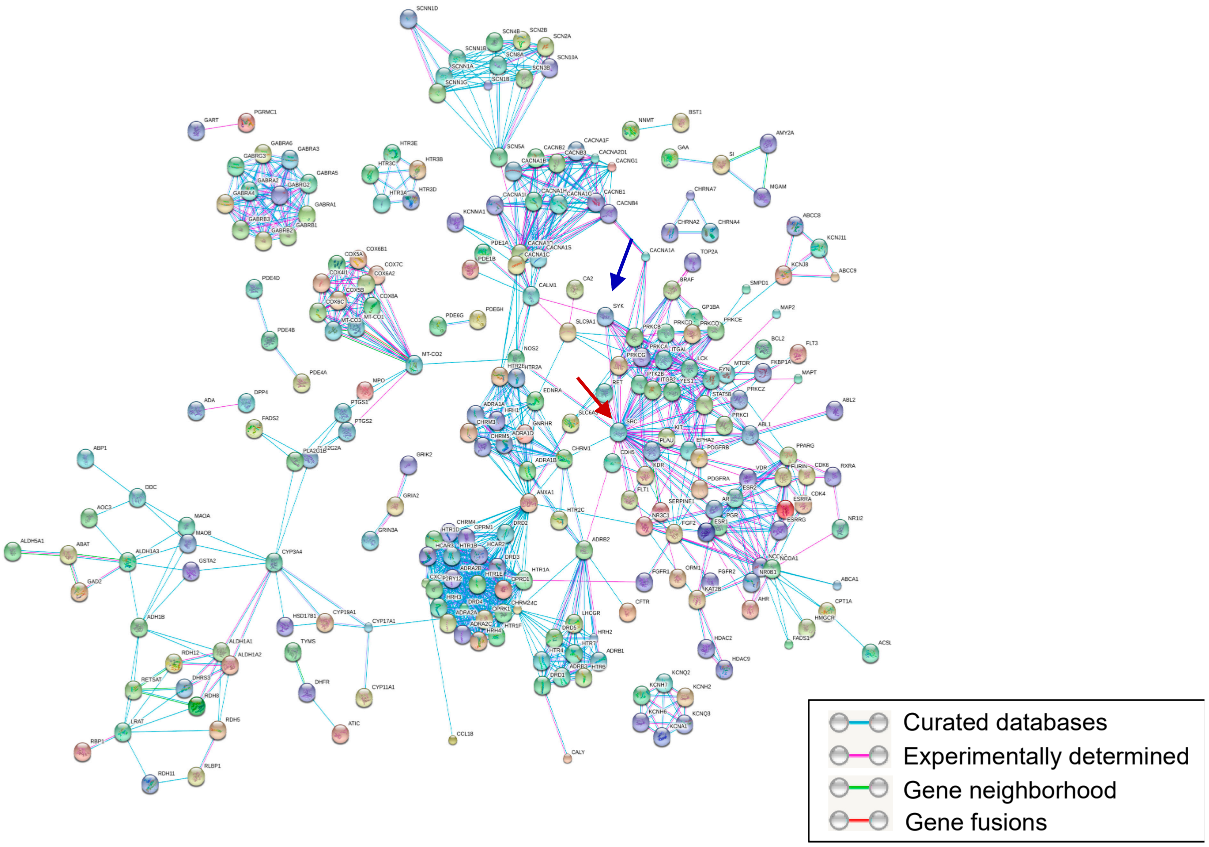

Figure S3. Drug target network. Resultant drug target protein-protein interaction (PPI) network from prioritized drug candidates using PPI interaction data from the STRING database (v 10.0). Only high confidence interaction scores (0.700 and above) from experiments, databases, neighborhood and gene fusion sources were included in the final network. Interaction types are indicated by edge color. Red arrow: SRC protein; blue arrow: SYK protein.

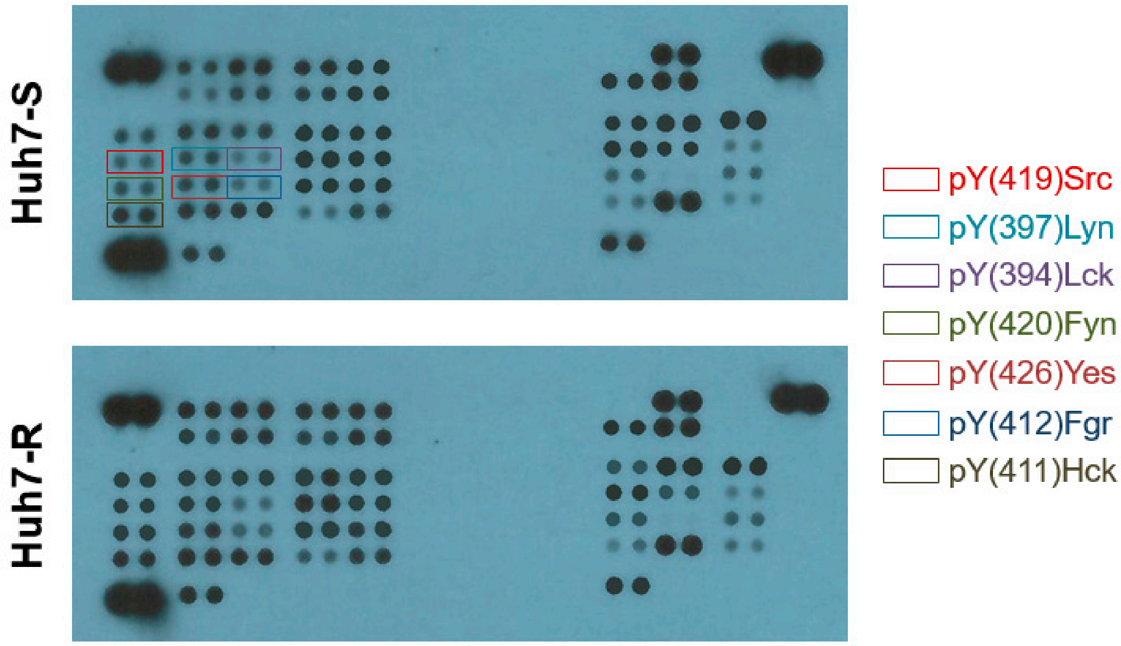

Figure S4. Dot blots of Proteome Profiler Human Phospho-Kinase Array.

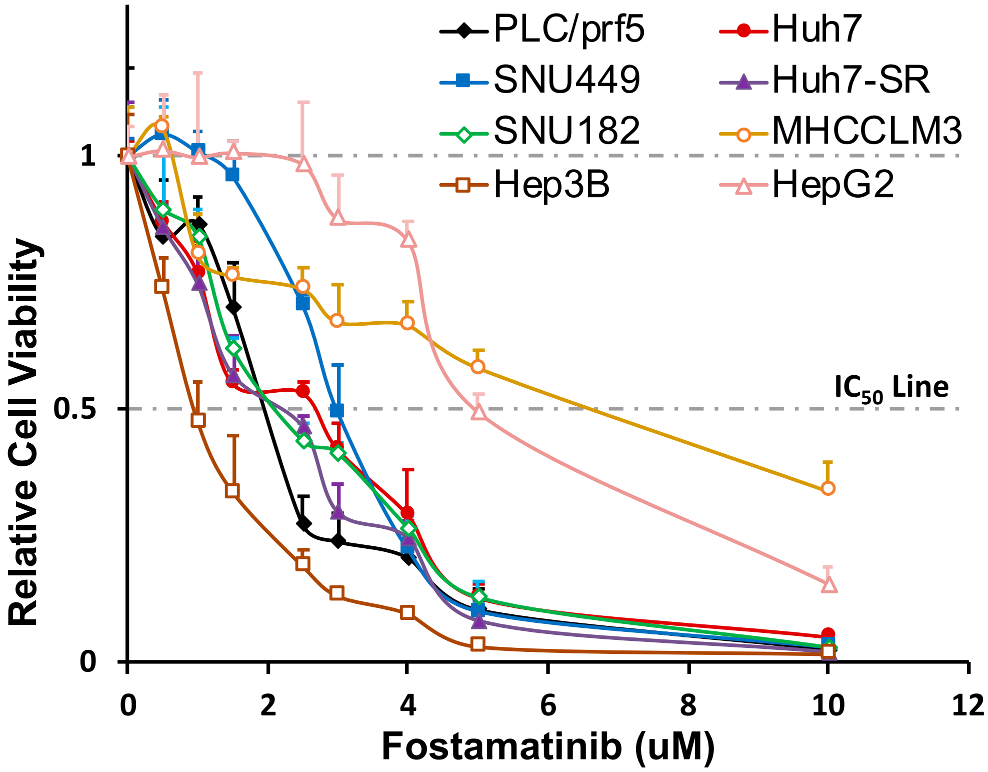

Figure S5. Fostamatinib dose response curve for HCC cell lines. Fold-change in cell viability of HCC cells treated with increasing concentrations of fostamatinib, as measured 48 hours post-treatment using an ATP assay (CellTiter-Glo).

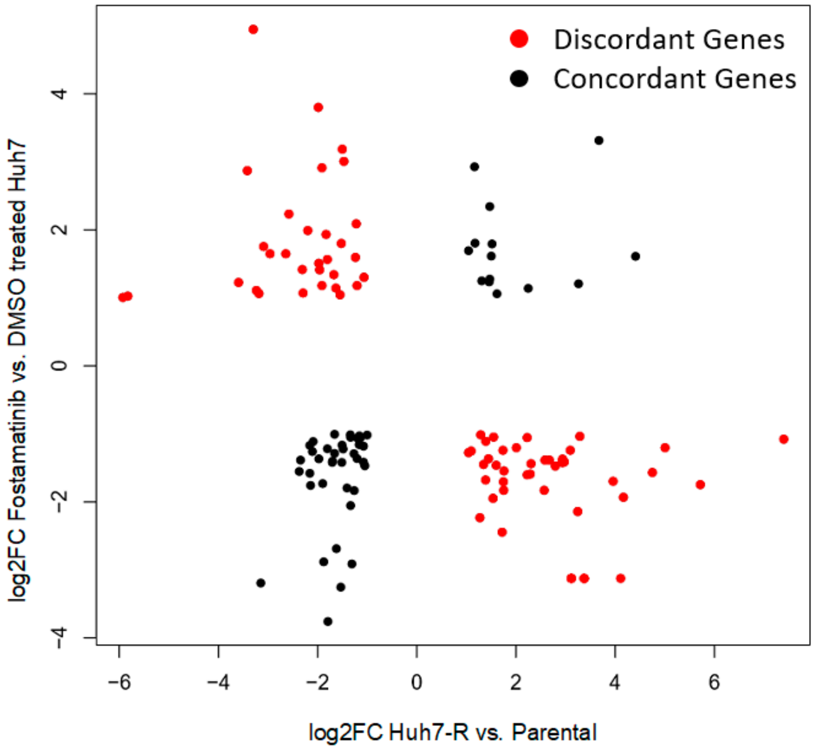

Figure S6. Correlation plot of gene expression log2 fold change between Huh7 sorafenib resistance (Huh7-R) signature and fostamatinib-treated Huh7 cells.

© 2020 by the authors. Licensee MDPI, Basel, Switzerland. This article is an open access article distributed under the terms and conditions of the Creative Commons Attribution (CC BY) license (http://creativecommons.org/licenses/by/4.0/).
