# Supplementary material for: Transcriptomics-Based Drug Repurposing Approach Identifies Novel Drugs against Sorafenib-Resistant Hepatocellular Carcinoma
Source: Cancers (Basel). 2020 Sep 23;12(10):2730. doi: 10.3390/cancers12102730 (PMC7598246; doi:10.3390/cancers12102730)
Supplement: Supplementary file 1 [file cancers-12-02730-s001.zip › Figure S7-Uncropped Western Blots.pdf]

Gel1 Huh7 and Huh7SR cell treat w/ DMSO or FOS

w/ phospho antibodies

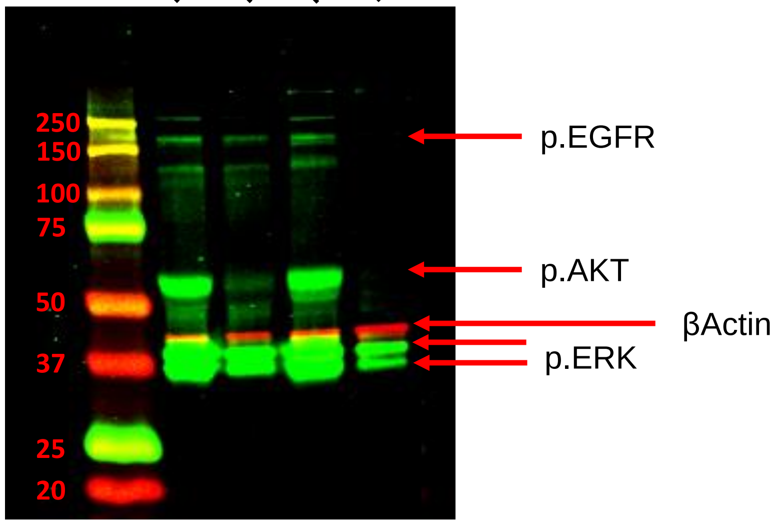

p.EGFR 100 : 12 : 110 : 7
p.AKT 100 : 11 : 103 : 7
p.ERK* 100 : 17 : 79 : 8

* combined signal of double bands

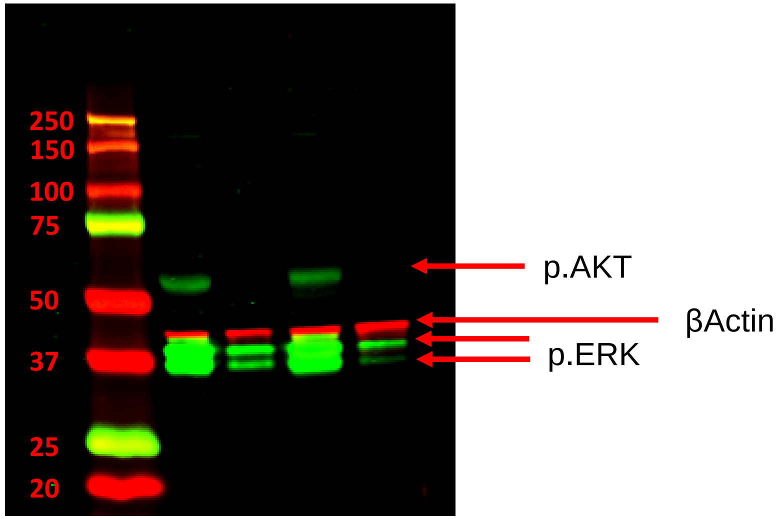

w/ total antibodies

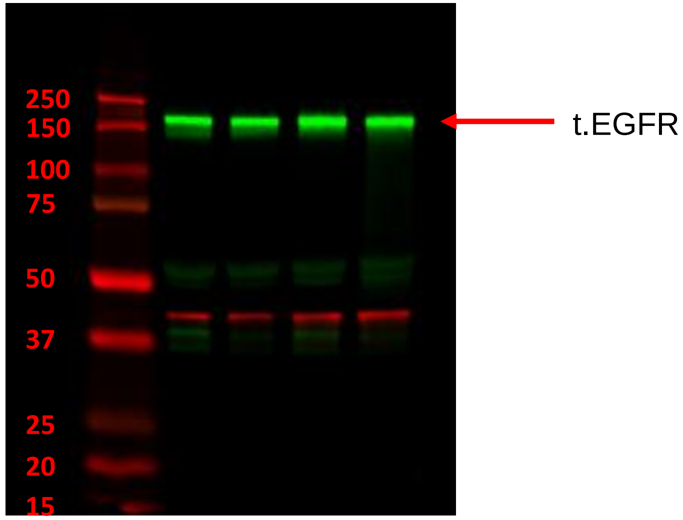

t.EGFR 100 : 104 : 125 : 121

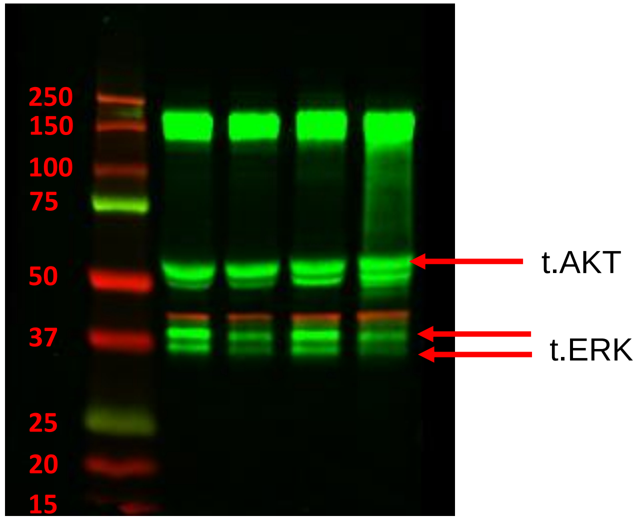

t.AKT 100 : 100 : 104 : 99
t.ERK * 100 : 42 : 90 : 37

* combined signal of double bands

Gel2 Huh7 and Huh7SR cell treat w/ DMSO or FOS

w/ phospho antibodies

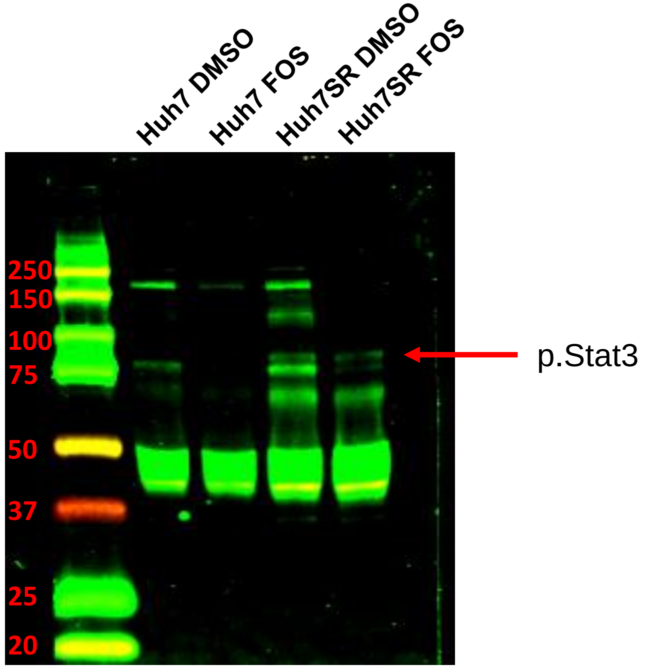

p.Stat3 100 : 12 : 255 : 49

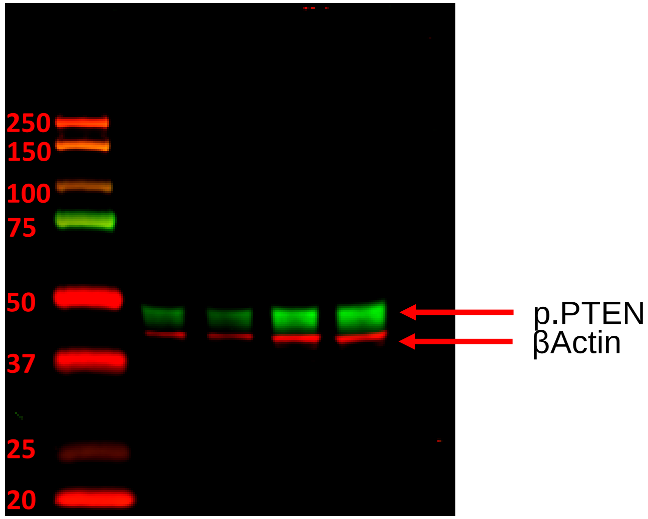

p.PTEN 100 : 101 : 216 : 213

w/ total antibodies

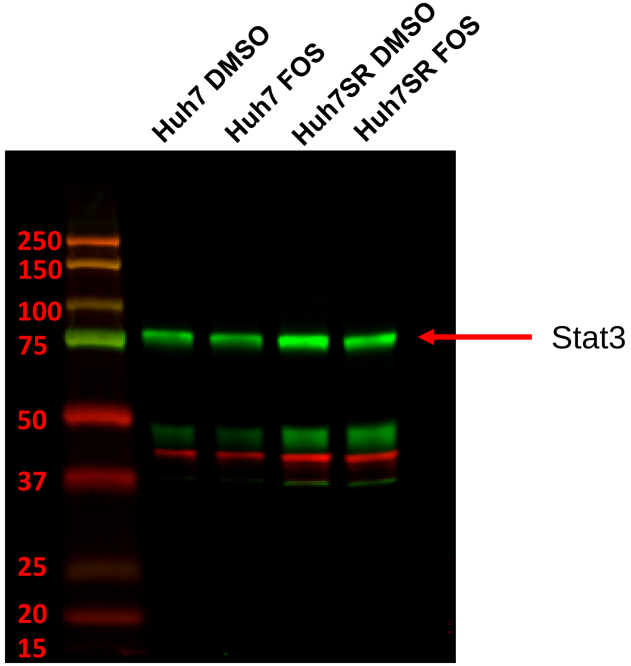

t.Stat3 100 : 97 : 175 : 141

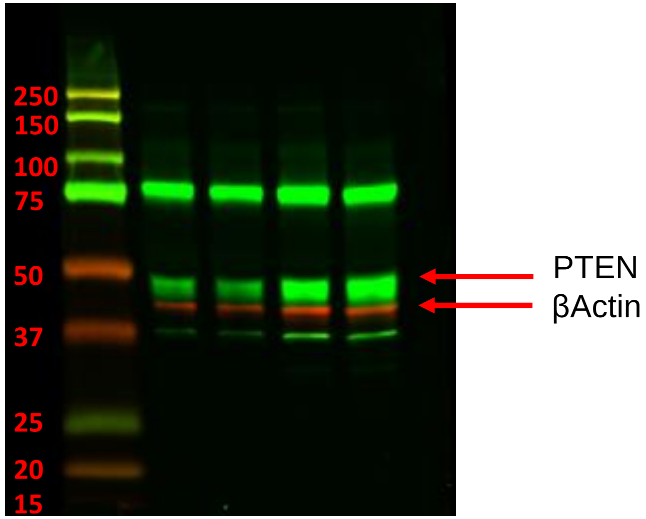

t.PTEN 100 : 106 : 252 : 283

Gel3 Huh7 and Huh7SR cell treat w/ DMSO or FOS

w/ phospho antibodies

Huh7 DMSO | Huh7 FOS | Huh7SR DMSO | Huh7SR FOS

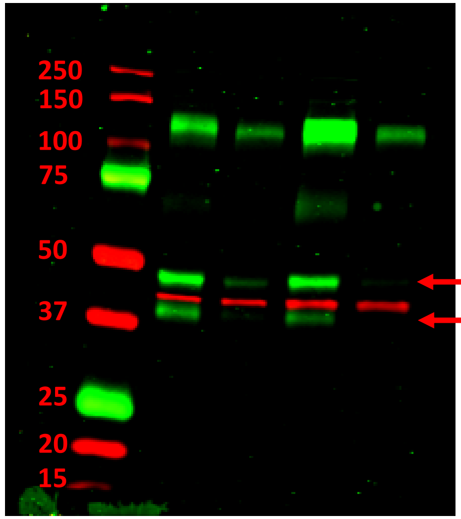

p.JNK

p.JNK* 100 : 20 : 88 : 12 *** combined signal of double bands**

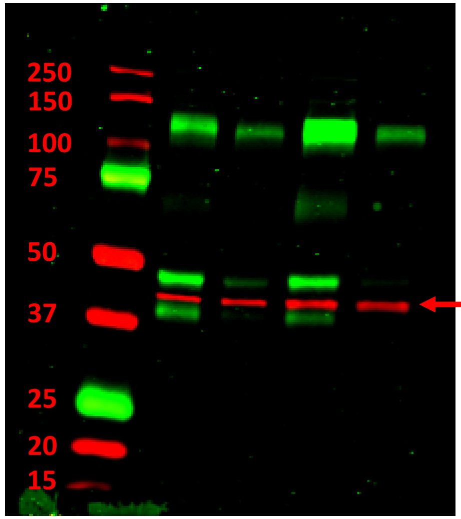

βActin

w/ total antibodies

Huh7 DMSO | Huh7 FOS | Huh7SR DMSO | Huh7SR FOS

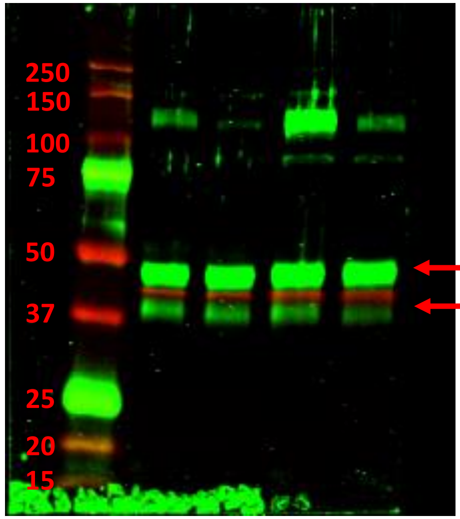

t.JNK(high)

t.JNK* 100 : 20 : 88 : 12 *** combined signal of double bands**

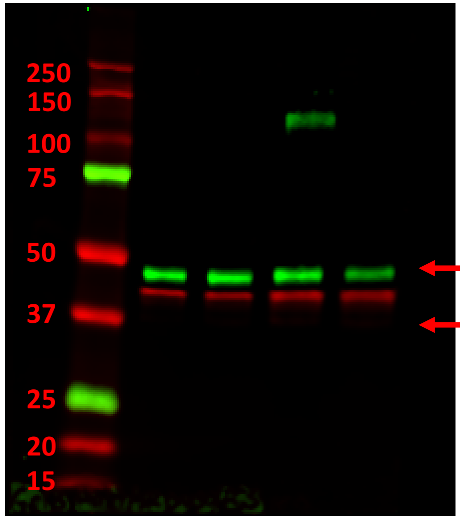

t.JNK(low)
